# The impact of vildagliptin on the daily glucose profile and coronary plaque stability in impaired glucose tolerance patients with coronary artery disease: VOGUE—A multicenter randomized controlled trial

**DOI:** 10.1186/s12872-021-01902-0

**Published:** 2021-02-15

**Authors:** Hiroyuki Yamamoto, Akihide Konishi, Toshiro Shinke, Hiromasa Otake, Masaru Kuroda, Tsuyoshi Osue, Takahiro Sawada, Tomofumi Takaya, Hiroya Kawai, Naoko Hashimoto, Takeshi Ohara, Yushi Hirota, Kazuhiko Sakaguchi, Takashi Omori, Wataru Ogawa, Ken-ichi Hirata

**Affiliations:** 1 Division of Cardiovascular Medicine, Department of Internal Medicine, Kobe University Graduate School of Medicine, Kobe, Japan; 2 Kobe University Hospital Clinical & Translational Research Center, Kobe, Japan; 3 Division of Cardiology, Department of Medicine, Showa University School of Medicine, 1-5-8 Hatanodai, Shinagawa-ku, Tokyo, 142-8555 Japan; 4 Division of Cardiovascular Medicine, Department of Internal Medicine, Hyogo Heart and Brain Center, Himeji, Japan; 5 Division of Diabetes and Endocrinology, Department of Internal Medicine, Hyogo Heart and Brain Center, Himeji, Japan; 6 Division of Diabetes and Endocrinology, Department of Internal Medicine, Kobe University Graduate School of Medicine, Kobe, Japan

**Keywords:** Vildagliptin, Fibrous cap thickness, Mean amplitude of glycemic excursion, Impaired glucose tolerance

## Abstract

**Background:**

The impact of reduction in glycemic excursion on coronary plaques remains unknown. This study aimed to elucidate whether a dipeptidyl peptidase 4 inhibitor could reduce the glycemic excursion and stabilize the coronary plaques compared with conventional management in coronary artery disease (CAD) patients with impaired glucose tolerance (IGT).

**Methods:**

This was a multicenter, randomized controlled trial including CAD patients with IGT under lipid-lowering therapy receiving either vildagliptin (50 mg once a day) or no medication (control group) regarding glycemic treatment. The primary endpoint was changes in the minimum fibrous cap thickness and lipid arc in non-significant native coronary plaques detected by optical coherence tomography at 6 months after intervention. Glycemic variability expressed as the mean amplitude of glycemic excursion (MAGE) measured with a continuous glucose monitoring system was evaluated before and 6 months after intervention.

**Results:**

A total of 20 participants with 47 lesions were allocated to either the vildagliptin group (10 participants, 22 lesions) or the control group (10 participants, 25 lesions). The adjusted difference of mean changes between the groups was − 18.8 mg/dl (95% confidence interval, − 30.8 to − 6.8) (*p* = 0.0064) for the MAGE (vildagliptin, − 20.1 ± 18.0 mg/dl vs. control, 2.6 ± 12.7 mg/dl), − 22.8° (− 40.6° to − 5.1°) (*p* = 0.0012) for the mean lipid arc (vildagliptin, − 9.0° ± 25.5° vs. control, 15.8° ± 16.8°), and 42.7 μm (15.3 to 70.1 μm) (*p* = 0.0022) for the minimum fibrous cap thickness (vildagliptin, 35.7 ± 50.8 μm vs. control, − 15.1 ± 25.2 μm).

**Conclusions:**

Vildagliptin could reduce the MAGE at 6 months and may be associated with the decreased lipid arc and increased minimum FCT of the coronary plaques in CAD patients with IGT as compared with the control group. These findings may represent its potential stabilization effect on coronary plaques, which are characteristic in this patient subset.

*Trial registration* Registered in the UMIN clinical trial registry (UMIN000008620), *Name of the registry: *VOGUE trial, *Date of registration: *Aug 6, 2012, *URL*: https://upload.umin.ac.jp/cgi-open-bin/ctr/ctr_view.cgi?recptno=R000010058

## Background

Accumulating evidence has revealed that treating dyslipidemia can reduce cardiovascular events; as such lipid-management with statins is used worldwide in the primary and secondary prevention of coronary artery disease (CAD). However, statin administration for the management of dyslipidemia has been reported to achieve only a 30% reduction in the risk of future cardiovascular events [1]. Thus, further management for the residual CAD risk apart from dyslipidemia is needed. Meanwhile, the clinical effect of abnormal glucose metabolism including diabetes has been known as an important treatable target for improving CAD prognosis. The number of recent reports on the postprandial blood glucose status contributing to the development of atherosclerosis has increased; moreover, a prospective randomized controlled trial on the management of impaired glucose tolerance (IGT) has revealed that larger postprandial glucose excursion accelerates atherosclerosis formation while improving postprandial hyperglycemic state prevents atherosclerosis progression [2–4]. Moreover, it has been well known that the 2-h postprandial hyperglycemic state assessed by oral glucose tolerance test (OGTT) is strongly associated with cardiovascular disease and an increased risk of death [5, 6]. Furthermore, we previously reported that glycemic variability assessed by continuous glucose monitoring (CGM) system may impact the accelerating plaque vulnerability representing lipid-rich atheroma with thinning fibrous cap detected by optical coherence tomography (OCT), a high-resolution intravascular imaging modality, in stable lipid-controlled CAD patients [7]. However, whether interventions against glycemic variability may improve coronary plaque characteristics in lipid-controlled CAD patients with IGT remains unknown.

Vildagliptin, an oral anti-hyperglycemic drug included in the class of dipeptidyl peptidase-4 (DPP-4) inhibitors, is widely used for type 2 diabetes [8]. A recent clinical trial has revealed that the reduction in daily glycemic variability by vildagliptin was superior to that by sitagliptin in Japanese patients with type 2 diabetes [9]. Furthermore, a previous double-blind randomized parallel-group study has revealed that the use of vildagliptin in patients with IGT achieved a 32% reduction in postprandial glucose levels without the occurrence of hypoglycemic events [10].

This study aimed to investigate the effect of vildagliptin on fibrous thickness expressed as plaque vulnerability of coronary plaques detected by OCT in CAD patients with IGT.

## Methods

### Study design

We conducted a multicenter, open-label, randomized controlled trial at two institutes in Japan: the Kobe University Hospital and Hyogo Brain and Heart Center. The study was conducted between September 1, 2012 and March 30, 2019. The ethical review board of each institution approved the protocol of the study at Kobe University Hospital and Hyogo Brain and Heart Center, respectively. Written informed consent to participate in the study was obtained from all patients. This study was registered to the UMIN clinical trial registry (UMIN000008620). This manuscript adheres to the CONSORT guidelines.

### Participants

Patients included in this study satisfied the following criteria: (1) scheduled to undergo percutaneous coronary intervention (PCI) for stable CAD with an "untreated IGT" defined as "2-h plasma/serum glucose level: 140 to 199 mg/dL in a 75 g OGTT", (2) under lipid-lowering management; low-density lipoprotein cholesterol < 120 mg/dl with statin administration, and < 100 mg/dl without statin administration, (3) between 20 and 80 years old, and 4) having provided written informed consent for participation in the study. Stable CAD was defined as a clinical syndrome characterized by effort chest discomfort, including shoulder or back pain, which could be relieved by rest or after nitroglycerin use, or a clinical syndrome characterized by ischemic signs on examination in asymptomatic patients [11]. Patients meeting any of the following conditions were excluded: (1) previously diagnosed as type-1 or type-2 diabetes, (2) severe liver or renal dysfunction, (3) severe heart failure classified as New York Heart Association stage III or IV, (4) malignancies or other diseases with poor prognosis, (5) pregnant women and those planning to get pregnant, and (6) judged as ineligible by clinical investigators.

### Treatment protocol

Patients who consented to participate and fulfilled the inclusion criteria were included in this study; PCI was performed in all participants to restore culprit lesions, which were defined as significant coronary lesions of an angiographical stenosis diameter of ≥ 70% with inducible ischemia as diagnosed by either an invasive or a non-invasive myocardial stress test. Participants were randomly divided into those receiving 50 mg vildagliptin once a day for at least 6 months (vildagliptin group) or diet and exercise therapy (control group). Other oral hypoglycemic drugs as for combination treatment were prohibited during the study period. The up titration and addition of lipid improving agents such as statin preparations, fibrate preparations, and eicosapentaenoic acid were prohibited as well. Coronary angiography, OCT, and CGM were conducted before and 6 months after PCI.

### CGM system

CGM was performed prior and 6 months after PCI. The daily glucose profile was analyzed using data obtained under stabilized conditions on the second and third day to avoid any bias caused by attaching and detaching the CGM sensor. Further, independent investigators blinded to other clinical data calculated the following variables measured using CGM analysis software (CareLink iPro, Medtronic, Northridge, CA): 24-h mean glucose levels, time in hyperglycemia (glucose levels > 140 mg/dl) / hypoglycemia (glucose levels < 70 mg/dl), and the mean amplitude of glycemic excursion (MAGE) [12]. All patients underwent CGM under nutritionally balanced meals; 25–28 kcal/kg of the ideal body weight, 60% carbohydrate, 15–20% protein, and 20–25% fat [7].

### OCT imaging

Angiographically non-significant coronary lesions (30% to 70% luminal narrowing via angiography) were enrolled to OCT assessment during index PCI procedure, and deemed to follow-up OCT assessment 6 months after PCI. Intravascular images were acquired with a frequency-domain OCT imaging system (ILUMIEN; St. Jude Medical Inc., St Paul, MN, USA). Investigators blinded to glycemic data used a 2.7-Fr OCT imaging catheter beyond lesions to be studied, and OCT images were obtained using automated pullback system during eliminating blood in the coronary artery by the injection of contrast media. All target plaques were non-culprit lesions, which form mild to moderate stenosis.

### OCT analysis

All OCT images were analyzed using Off-line Review Workstation by two independent investigators blinded to the angiographic and clinical findings. Using OCT examination, all plaques of interest were evaluated at every 1-mm interval throughout each lesion as follows: lipid length, lipid arc, calcification length, and calcification arc. The lipid length was measured on the longitudinal view and lipid arc was measured at each 1-mm cross-section, and the values were averaged [13]. The calcification consisting of a poor or heterogeneous signal region with a sharply delineated border [14] was also measured using the same method as in the lipidic parameters [7]. Fibrous cap was evaluated according to previous definition as a signal-rich homogenous layer overlying the lipid-rich plaque [15]. The minimum fibrous cap thickness (FCT) was defined as the average thickness after measuring three times the thinnest part of the fibrous cap [16].

### Outcomes

In this trial protocol, the primary endpoint was changes in coronary plaque characteristics, such as the minimum FCT and lipid arc detected by OCT between baseline and 6 months after intervention, and those in the MAGE.

The following OCT parameters were determined for analysis: the minimum lumen area, lipid length, lipid mean arc, and minimum FCT for quantitative variables. As outcomes of glycemic metabolic variables, the MAGE, time in hyperglycemia/ hypoglycemia, and mean, maximum, and minimum blood glucose levels were measured. As clinical outcome, cardiac death, myocardial infarction (MI), cerebral infarction, target lesion revascularization (TLR), and target vessel revascularization (TVR) were set.

### Sample size

The sample size could not be calculated based on statistical power because of the novel nature of this research. We determined the sample size after reviewing the literature on similar studies, where a sample size of 9 to 19 individuals per group was deemed necessary to investigate the effects of vildagliptin on glucose fluctuation, oxidative stress, and endothelial function [17–19]. Based on these studies and after allowing for the possibility of a 30% dropout rate, the sample size for this study was calculated as 25 individuals per group.

## Randomization

### Sequence generation

Participants were randomly assigned to either the vildagliptin or the control group with 1:1 allocation by permuted-block randomization using an excel-based allocation system with stratification.

### Allocation concealment mechanism

Randomization was performed by an allocation stuff member in the Kobe University Hospital who was not a clinician.

### Implementation

After a participant who consented to participate and fulfilled the inclusion criteria was enrolled in the study, randomization was performed by the allocation stuff.

### Blinding

Participants and investigators were not masked to the allocation; however, both OCT images and CGM data were analyzed in a blinded manner.

### Statistical methods

The data set consisted of all participants enrolled in this study. For the participant baseline data in analysis population, summary statistics are presented by group. The categorical counts and proportion were calculated for nominal variables; number of participants, mean, standard deviation, minimum value, median, and maximum value for continuous variables. For inter-group comparison of nominal and continuous variables, Fisher’s exact test and Wilcoxon’s rank sum test were used, respectively.

For continuous OCT parameters and glycemic metabolic variables, the adjusted mean difference of the means for the change between baseline and 6 months after intervention between the vildagliptin and control groups was estimated, whereas, for binomial OCT parameters, the adjusted odds ratio was estimated, along with 95% confidence interval (CI). Since the observed values of OCT parameters were measured for the lesions, the above estimation was performed with the generalized estimate equation under the assumption of the intra-subject correlation structure of compound symmetry. The least square method was used for the glycemic metabolic variables because of an observed value per person. For both estimations, as independent variables, the baseline value of each parameter along with group variables were included in the statistical model.

For clinical outcomes, risk difference and 95% CI were estimated. A statistical test between the two groups based on z-statistics for OCT parameters, t-statistics for the glycemic metabolic variables, and Fisher’s exact test for the clinical outcome were performed and both sides *p* values were reported. We did not conduct any adjustment for multiplicity for statistical test, as concrete primary and secondary endpoint were not specified in the protocol. All statistical analyses were conducted using SAS software (version 9.4, SAS Institute, Cary, NC).

## Results

### Participants

**Fig. 1:**
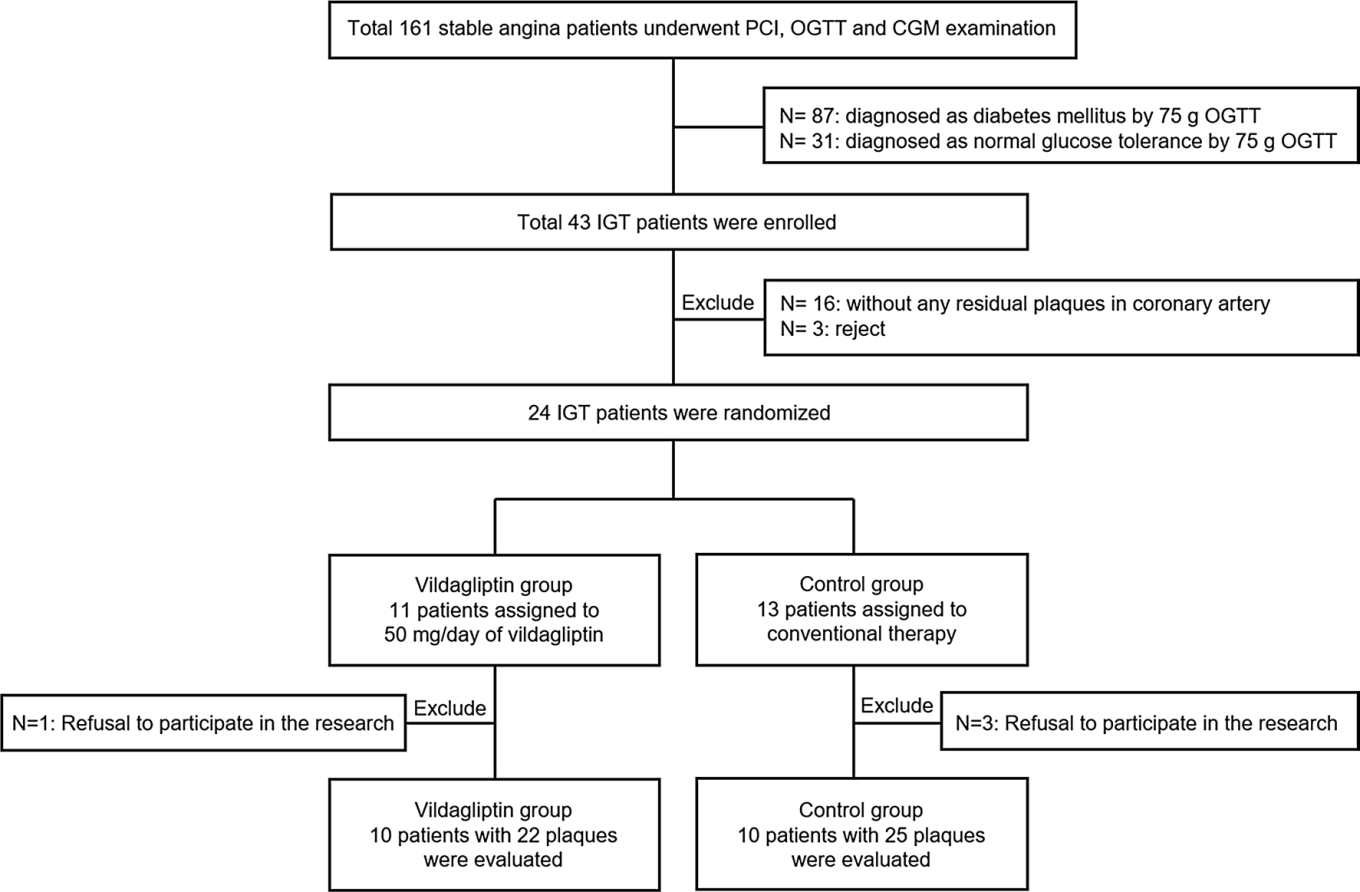
Participant flow diagram of the present study. OCT = optical coherence tomography, PCI = percutaneous coronary intervention, OGTT = oral glucose tolerance test, CGM = continuous glucose monitoring, IGT = impaired glucose tolerance

Figure 1 shows the participant flow diagram of the study. Of a total 24 patients included in this study, 11 allocated in the vildagliptin group and 13 in the control group, 4 patients (vildagliptin group: n = 1, control group: n = 3) were excluded because they refused to participate in the follow-up study. Finally, 20 patients (vildagliptin group: n = 10, control group: n = 10) were followed up completely.

All 20 participants were followed up for 6 months, and the outcomes of glycemic metabolic variables and clinical outcomes were measured. However, one participant of the control group did not perform the 6-month OCT because of poor renal dysfunction.

### Recruitment

This study started on September 1, 2012, and the planned follow-up period for all participants was at least 6 months. However, the study was terminated on March 30, 2019 because of delays in the enrollment towing to the scarcity of IGT patients who have already developed significant CAD.

### Baseline data

Table 1 shows the baseline demographic and clinical characteristics of the participants. The median (range) of the MAGE was 67.7 mg/dl (50.3 to 88.1 mg/dl) in the vildagliptin group and 61.8 mg/dl (39.5 to 72.1 mg/dl) in the control group (*p* = 0.307).

**Table 1:** Baseline patient and lesion characteristics

Variables	Vildagliptin group (n = 10)	Control group (n = 10)	*p* Value
Men, %	100	80	0.474
Age, years	65.9 ± 10.6	68.3 ± 8.1	0.820
BMI, kg/m^2^	24.0 ± 4.0	24.0 ± 4.5	0.909
Smoker, %	70	40	0.133
Familial history, %	10	10	1.000
Stable angina, %	100.0	100.0	
Revascularization vessel, %			0.409
Right coronary artery, %	31.8	34.6	
Left main coronary trunk, %	18.2	7.7	
Left anterior descending coronary artery, %	31.8	34.6	
Left circumflex coronary artery, %	18.2	23.1	
Medication, %			
Beta blocker, %	70.0	50.0	0.649
ACE-I/ARB, %	50.0	50.0	1.000
Statin, %	90.0	100.0	1.000
Other oral DM drugs, % (except DPP-4 inhibitors)	0.0	0.0	
Insulin, %	0.0	0.0	
Prasugrel, %	40.0	60.0	0.656
Clopidogrel, %	60.0	40.0	0.656
Aspirin, %	100.0	100.0	0
Laboratory data			
LDL cholesterol, mg/dl	84.0 ± 14.3	82.9 ± 27	0.791
HDL cholesterol, mg/dl	47.4 ± 14.8	41.5 ± 9.8	0.289
Triglyceride, mg/dl	118.4 ± 52.1	131.6 ± 77.9	0.939
Creatinine, mg/dl	0.8 ± 0.1	0.9 ± 0.3	1.000
1,5 AG, μg/ml	19.9 ± 4.1	22.0 ± 4.1	0.377
HbA1c (NGSP), %	5.9 ± 0.3	5.9 ± 0.4	0.845
HOMA R	1.5 ± 5.7	1.5 ± 0.6	0.909
CRP, mg/dl	0.2 ± 0.4	0.2 ± 0.2	0.879
75 g OGTT			
Fast glucose, mg/dl	97.2 ± 9.0	94.2 ± 11.9	0.471
2-h glucose, mg/dl	185.7 ± 36.6	169.5 ± 20.5	0.405
Fast IRI, μU/ml	6.6 ± 3.5	6.3 ± 2.7	1.000
2-h IRI, μU/ml	114.1 ± 80.3	101.3 ± 75.6	0.791

Mean ± standard deviation (n) for continuous variables; frequency count (%) for categorical variables 1,5 AG = 1,5 anhydroglucitol; 75 g OGTT = 75 g oral glucose tolerance test; ACE-I = angiotensin converting enzyme-inhibitor; ARB = angiotensin II receptor blocker; BMI = body mass index; CRP = C-reactive protein; DM = diabetes mellitus; DPP-4 = dipeptidyl peptidase-4; HbA1c = glycated hemoglobin; HDL = high-density lipoprotein; HOMA R = homeostasis model assessment ratio; IRI = immunoreactive insulin; LDL = low-density lipoprotein; NGSP = national glycohemoglobin standardization program

### Numbers of analyzed participants

Numbers of analysis unit were 10 individuals per group for outcomes of glycemic metabolic variables and clinical outcomes, whereas 22 lesions in the vildagliptin group and 25 lesions in the control group for the evaluation of plaque characteristics.

### Outcome measures

**Fig. 2:**
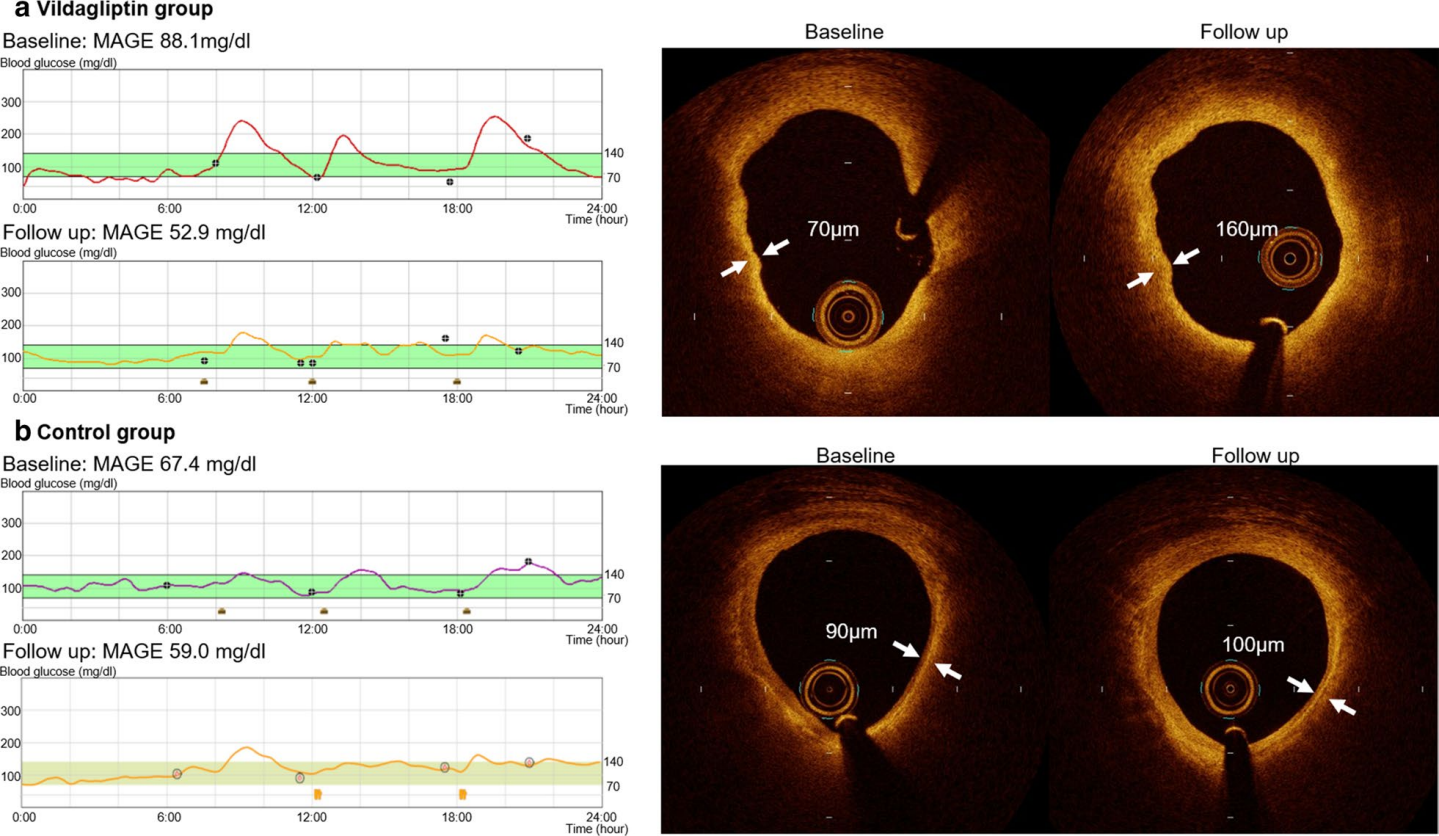
Representative cases of the mean amplitude of glucose excursion (MAGE) and minimum fibrous cap thickness (FCT) in the vildagliptin and control groups: **a** Representative case of the vildagliptin group. The MAGE was dramatically reduced from 88.1 mg/dl at baseline to 52.9 mg/dl at follow-up. Optical coherence tomography (OCT) imaging showed that the thinnest part of FCT on lipid-rich plaque was improved from 70 µm to 160 µg (lengths between white allows showing the minimum FCT). **b** Representative case of the control group. The MAGE was almost unchanged from 67.4 mg/dl at baseline to 59.0 mg/dl at follow-up. OCT imaging showed that the minimum FCT remained thin and virtually unchanged (90 µm at baseline and 100 µg at follow-up)

For the glycemic metabolic variables, the adjusted mean difference of the changes between two groups are shown in Table 2. The mean change of the MAGE in the vildagliptin group was lower than that in the control group (vildagliptin: − 20.1 ± 18.0 mg/dl vs. control: 2.6 ± 12.7 mg/dl, *p* = 0.0064). The representative case of the MAGE in both groups is described in Fig. 2.

**Table 2:** Variables measured by the continuous glucose monitoring system and plaque characteristics detected by optical coherence tomography

Variables	Change in the vildagliptin group (6 months—baseline)	Change in the control group (6 months—baseline)	Difference between mean change		
			Difference	95% confidence interval	*p* Value
Variables measured by the continuous glucose monitoring system					
	(n = 10)	(n = 10)			
MAGE, mg/dl	− 20.2 ± 18.0	2.6 ± 12.7	− 18.8	− 30.8 to − 6.8	0.0064
Time in hyperglycemia, hours	− 7.9 ± 14.1	− 0.8 ± 10.2	− 3.7	− 11.4 to 3.9	0.350
Time in hypoglycemia, hours	− 0.8 ± 3.5	4.7 ± 15.4	− 6.1	− 15.3 to 3.1	0.209
Mean BS, mg/dl	− 3.3 ± 17.3	0.4 ± 13.3	0.3	− 9.5 to 10.0	0.960
Maximum BS, mg/dl	− 36.3 ± 65.8	1.2 ± 37.6	− 11.5	− 43.1 to 20.0	0.482
Minimum BS, mg/dl	5.5 ± 24.8	7.6 ± 23.1	4.8	− 10.3 to 20.0	0.538
HOMA R	0.6 ± 0.7	0.1 ± 1.0	0.5	− 0.3 to 1.3	0.243
Plaque characteristics detected by optical coherence tomography					
	(n = 22)	(n = 25)			
MLA, mm^2^	0.62 ± 1.94	− 0.29 ± 0.65	0.99	− 0.26 to 2.23	0.120
Lipid length, mm	− 0.10 ± 1.40	− 0.52 ± 2.37	0.03	− 1.28 to 1.34	0.963
Lipid arc, °	− 9.00 ± 25.57	15.87 ± 16.82	− 22.82	− 40.56 to − 5.09	0.017
Minimum FCT, μm	35.75 ± 50.80	− 15.19 ± 25.02	42.73	15.34 to 70.12	0.0022

Mean ± standard deviation (n) for continuous variables

Time in hyperglycemia was defined as the time when blood glucose levels were above 140 mg/dl. Time in hypoglycemia was defined as the time when blood glucose levels were under 70 mg/dl

BS = blood sugar; FCT = fibrous cap thickness; HOMA R = homeostasis model assessment ratio; MAGE = mean amplitude of glycemic excursion**;** MLA = minimum lumen area

For the OCT parameters, the mean difference of the changes between two groups for continuous variables and adjusted odds ratio for binomial variables are shown in Table 2. Lipid mean arc decreased in the vildagliptin group and increased in the control group (vildagliptin: − 9.0 ± 25.5° vs. control: 15.8 ± 16.8°, *p* = 0.0117). Moreover, the minimum FCT showed more than 40 μm increase in the vildagliptin group as compared with the control group (vildagliptin: 35.7 ± 50.8 μm vs. control: − 15.1 ± 25.2 μm, *p* = 0.0022). Figure 2 shows the representative case of the differences of the minimum FCT in both groups.

Table 3 shows the risk difference of the clinical outcomes. There was no occurrence of cardiac death, MI, or cerebral infarction in both groups. Although there were no cases of TLR and TVR in the vildagliptin group, one case of TLR and two of TVR were observed in the control group.

**Table 3:** Clinical outcomes

Variables	Vildagliptin group (n = 10)	Control group (n = 10)	*p* Value
Cardiac death, %	0	0	
Myocardial infarction, %	0	0	
Cerebral infarction, %	0	0	
Target lesion revascularization, %	0	10	1.000
Target vessel revascularization, %	0	20	0.473

## Discussion

In this study, we assessed the effect of vildagliptin in lipid-controlled CAD patients with IGT on non-culprit native coronary plaques detected by OCT. The major findings were as follows: (1) the absolute decrease in the MAGE was greater in the vildagliptin group than in the control group, and (2) the absolute increase in the minimum FCT and the absolute decrease in the lipid arc were higher in the vildagliptin group than in the control group.

It has been recently reported that glycemic variability such as postprandial hyperglycemia or hypoglycemia, which occur at an early stage of abnormal glucose tolerance, may be a significant factor aggravating the development of CAD, apart from dyslipidemia [2, 5]. Several studies have suggested that large glycemic variability may have stronger association with vascular injury compared with constant high blood glucose levels [20, 21]. Teraguchi et al. [22] reported that glycemic variability expressed as the MAGE was significantly associated with coronary plaque rupture in acute MI patients. These investigations suggest that poor management of daily glycemic variability could adversely affect the endothelial function promoting atherosclerosis progression and the advancement of plaque vulnerability, leading to fatal cardiovascular events. However, a relationship between reduction in glucose fluctuations and stabilization of coronary plaque has not been reported previously. This study is the first to report that vildagliptin could reduce glucose fluctuation and simultaneously stabilize coronary plaque morphology in patients with IGT.

Several reports have showed that DPP-4 inhibitors, including vildagliptin, may present in vitro anti-atherosclerotic and cardioprotective effects [23–25]. Kuramitsu S, et al. [26] reported that sitagliptin did not significantly reduce coronary plaque volume, but the percent change in lipid plaque volume significantly decreased in the sitagliptin group compared to receiving conventional therapy with diet and exercise (− 7.1 ± 21.5% vs. 15.6 ± 41.8%, *p* = 0.03) at 6-month follow-up; this finding suggests that sitagliptin has a potential to prevent coronary plaque progression in diabetic patients with acute coronary syndrome (ACS). In our study, the absolute increase in the minimum FCT and the absolute decrease in the lipid arc when treated by vildagliptin suggest that vildagliptin has a potential benefit to stabilize the plaque vulnerability even in thin-cap fibroatheroma (TCFA), which is considered as the cause of ACS. As a matter of fact, there were no TLR and TVR cases reported in the vildagliptin group; however, one TLR (10%) and two TVR (20%) cases were observed in the control group in this study. Moreover, further large studies are warranted to elucidate whether early intervention with vildagliptin can lead to better clinical outcomes in patients with IGT. However, it was beyond the scope of our study to precisely identify if plaque stabilization in the vildagliptin group was the result of a reduction in glucose fluctuation or because of atherosclerosis reduction promoted by the drug itself. On considering the effect of the MAGE on coronary plaque vulnerability, as reported by previous research, it could be speculated that coronary plaque stabilization was chiefly affected by the glycemic modification effects of the drug [4, 7, 17, 20]. Further research is necessary to elucidate the mechanism of the plaque stabilization in this setting.

## Limitations

This study has several limitations. First, the small sample size led to imprecise estimates of effect measures, differences, odds ratio, and risk differences. However, important outcomes of this study, such as the MAGE, mean lipid arc, and minimum FCT, tended to clearly differ between the two groups. Another limitation is that the definition of the primary endpoint described in the study protocol was obscure. We could not adjust multiplicity in the data analysis for outcomes because we did not know how many outcomes were used. Therefore, we did not state any statistical significance in this data analysis. Furthermore, we only assessed stable CAD patients. Therefore, patients with ACS were not included in this study because this population has multiple confounding factors, such as uncontrolled dyslipidemia, stress-induced hyperglycemia, and drastic interventional changes including diet and exercise or administration of optimal medications after admission. Further study is warranted to elucidate whether stabilizing glycemic variability might improve plaque vulnerability in ACS patients with IGT, because more vulnerable plaques might be observed in such patients. Moreover, we did not validate the OCT findings with histological examination in this study. Therefore, it is unclear whether vildagliptin could actually stabilize TCFA, which leads to fatal coronary events. Finally, the open-label study design may have yielded a placebo effect on the subjective data due to the active intervention. However, this may not have affected our results because the primary endpoints were objective parameters, which were independently evaluated by researchers blinded to the clinical data. Considering these limitations, more larger scale double-blinded controlled trials are necessary to confirm if reducing glucose fluctuation can stabilize plaque vulnerability and reduce TCFA.

## Conclusions

Vildagliptin could reduce the MAGE at 6 months and may be associated with the decreased lipid arc and increased minimum FCT of the coronary plaques in CAD patients with IGT as compared with the control group. These findings may represent its potential stabilization effect on coronary plaques, which are characteristic in this patient subset.

## Data Availability

The datasets used and/or analyzed during the current study are available from the corresponding author on reasonable request.

## Abbreviations

ACS: Acute coronary syndrome;
CAD: Coronary artery disease
CGM: Continuous glucose monitoring
DPP-4: Dipeptidyl peptidase-4
FCT: Fibrous cap thickness
IGT: Impaired glucose tolerance
MAGE: Mean amplitude of glycemic excursion
MI: Myocardial infarction
OCT: Optical coherence tomography
OGTT: Oral glucose tolerance test
PCI: Percutaneous coronary intervention
TCFA: Thin-cap fibroatheroma
TLR: Target lesion revascularization
TVR: Target vessel revascularization

## References

[1] Shepherd J, Cobbe SM, Ford I, Isles CG, Lorimer AR, MacFarlane PW (1995). Prevention of coronary heart disease with pravastatin in men with hypercholesterolemia. West of Scotland Coronary Prevention Study Group. N Engl J Med..

[2] Chiasson JL, Josse RG, Gomis R, Hanefeld M, Karasik A, Laakso M (2002). Acarbose for prevention of type 2 diabetes mellitus: the STOP-NIDDM randomised trial. Lancet.

[3] Ceriello A (2000). The post-prandial state and cardiovascular disease: relevance to diabetes mellitus. Diabetes Metab Res Rev.

[4] Kuroda M, Shinke T, Sakaguchi K, Otake H, Takaya T, Hirota Y (2015). Effect of daily glucose fluctuation on coronary plaque vulnerability in patients pre-treated with lipid-lowering therapy: a prospective observational study. JACC Cardiovasc Interv.

[5] Glucose tolerance and mortality: comparison of WHO and American Diabetes Association diagnostic criteria. The DECODE study group. European Diabetes Epidemiology Group. Diabetes Epidemiology: Collaborative analysis Of Diagnostic criteria in Europe. Lancet. 1999;354:617–21. 10466661

[6] Tominaga M, Eguchi H, Manaka H, Igarashi K, Kato T, Sekikawa A (1999). Impaired glucose tolerance is a risk factor for cardiovascular disease, but not impaired fasting glucose. Funagata Diabet Stud Diabet Care.

[7] Kuroda M, Shinke T, Sakaguchi K, Otake H, Takaya T, Hirota Y (2015). Association between daily glucose fluctuation and coronary plaque properties in patients receiving adequate lipid-lowering therapy assessed by continuous glucose monitoring and optical coherence tomography. Cardiovasc Diabetol.

[8] Iwamoto Y, Kashiwagi A, Yamada N, Terao S, Mimori N, Suzuki M (2010). Efficacy and safety of vildagliptin and voglibose in Japanese patients with type 2 diabetes: a 12-week, randomized, double-blind, active-controlled study. Diabetes Obes Metab.

[9] Nomoto H, Kimachi K, Miyoshi H, Kameda H, Cho KY, Nakamura A (2017). Effects of 50 mg vildagliptin twice daily vs 50 mg sitagliptin once daily on blood glucose fluctuations evaluated by long-term self-monitoring of blood glucose. Endocr J..

[10] Rosenstock J, Foley JE, Rendell M, Landin-Olsson M, Holst JJ, Deacon CF (2008). Effects of the dipeptidyl peptidase-IV inhibitor vildagliptin on incretin hormones, islet function, and postprandial glycemia in subjects with impaired glucose tolerance. Diabetes Care.

[11] Montalescot G, Sechtem U, Achenbach S, Andreotti F, Arden C, Budaj A (2013). 2013 ESC guidelines on the management of stable coronary artery disease: the task force on the management of stable coronary artery disease of the European Society of Cardiology. Eur Heart J.

[12] Service FJ, Molnar GD, Rosevear JW, Ackerman E, Gatewood LC, Taylor WF (1970). Mean amplitude of glycemic excursions, a measure of diabetic instability. Diabetes.

[13] Yabushita H, Bouma BE, Houser SL, Aretz HT, Jang IK, Schlendorf KH (2002). Characterization of human atherosclerosis by optical coherence tomography. Circulation.

[14] Tearney GJ, Regar E, Akasaka T, Adriaenssens T, Barlis P, Bezerra HG (2012). Consensus standards for acquisition, measurement, and reporting of intravascular optical coherence tomography studies: a report from the International Working Group for Intravascular Optical Coherence Tomography Standardization and Validation. J Am Coll Cardiol.

[15] Burgmaier M, Hellmich M, Marx N, Reith S (2014). A score to quantify coronary plaque vulnerability in high-risk patients with type 2 diabetes: an optical coherence tomography study. Cardiovasc Diabetol.

[16] Kume T, Akasaka T, Kawamoto T, Okura H, Watanabe N, Toyota E (2006). Measurement of the thickness of the fibrous cap by optical coherence tomography. Am Heart J.

[17] Marfella R, Barbieri M, Grella R, Rizzo MR, Nicoletti GF, Paolisso G (2010). Effects of vildagliptin twice daily vs sitagliptin once daily on 24-hour acute glucose fluctuations. J Diabetes Complications..

[18] Rizzo MR, Barbieri M, Marfella R, Paolisso G (2012). Reduction of oxidative stress and inflammation by blunting daily acute glucose fluctuations in patients with type 2 diabetes: role of dipeptidyl peptidase-IV inhibition. Diabetes Care.

[19] van Poppel PC, Netea MG, Smits P, Tack CJ (2011). Vildagliptin improves endothelium-dependent vasodilatation in type 2 diabetes. Diabetes Care.

[20] Monnier L, Mas E, Ginet C, Michel F, Villon L, Cristol JP (2006). Activation of oxidative stress by acute glucose fluctuations compared with sustained chronic hyperglycemia in patients with type 2 diabetes. JAMA.

[21] Mita T, Otsuka A, Azuma K, Uchida T, Ogihara T, Fujitani Y (2007). Swings in blood glucose levels accelerate atherogenesis in apolipoprotein E-deficient mice. Biochem Biophys Res Commun.

[22] Teraguchi I, Imanishi T, Ozaki Y, Tanimoto T, Orii M, Shiono Y (2014). Impact of glucose fluctuation and monocyte subsets on coronary plaque rupture. Nutr Metab Cardiovasc Dis.

[23] Dei Cas A, Spigoni V, Cito M, Aldigeri R, Ridolfi V, Marchesi E (2017). Vildagliptin, but not glibenclamide, increases circulating endothelial progenitor cell number: a 12-month randomized controlled trial in patients with type 2 diabetes. Cardiovasc Diabetol.

[24] Drucker DJ, Nauck MA (2006). The incretin system: glucagon-like peptide-1 receptor agonists and dipeptidyl peptidase-4 inhibitors in type 2 diabetes. Lancet.

[25] Matsubara J, Sugiyama S, Sugamura K, Nakamura T, Fujiwara Y, Akiyama E (2012). A dipeptidyl peptidase-4 inhibitor, des-fluoro-sitagliptin, improves endothelial function and reduces atherosclerotic lesion formation in apolipoprotein E-deficient mice. J Am Coll Cardiol.

[26] Kuramitsu S, Miyauchi K, Yokoi H, Suwa S, Nishizaki Y, Yokoyama T (2017). Effect of sitagliptin on plaque changes in coronary artery following acute coronary syndrome in diabetic patients: The ESPECIAL-ACS study. J Cardiol.

